# What Am I Drinking? Vision Modulates the Perceived Flavor of Drinks, but No Evidence of Flavor Altering Color Perception in a Mixed Reality Paradigm

**DOI:** 10.3389/fpsyg.2021.641069

**Published:** 2021-07-20

**Authors:** Lorena Stäger, Marte Roel Lesur, Bigna Lenggenhager

**Affiliations:** Department of Psychology, Cognitive Neuropsychology, University of Zurich, Zurich, Switzerland

**Keywords:** visual and flavor interactions, cross modulations, bilateral cross modulations, multisensory perception, virtual reality

## Abstract

It is well established that vision, and in particular color, may modulate our experience of flavor. Such cross-modal correspondences have been argued to be bilateral, in the sense that one modality can modulate the other and vice versa. However, the amount of literature assessing how vision modulates flavor is remarkably larger than that directly assessing how flavor might modulate vision. This is more exaggerated in the context of cross-modal contrasts (when the expectancy in one modality contrasts the experience through another modality). Here, using an embodied mixed reality setup in which participants saw a liquid while ingesting a contrasting one, we assessed both how vision might modulate basic dimensions of flavor perception and how the flavor of the ingested liquid might alter the perceived color of the seen drink. We replicated findings showing the modulation of flavor perception by vision but found no evidence of flavor modulating color perception. These results are discussed in regard to recent accounts of multisensory integration in the context of visual modulations of flavor and bilateral cross-modulations. Our findings might be important as a step in understanding bilateral visual and flavor cross-modulations (or the lack of them) and might inform developments using embodied mixed reality technologies.

## Introduction

The quote “we eat with our eyes first” communicates the popular notion that vision has an impact in our perception of flavor, an idea confirmed through ingenious experimental setups and often used for marketing strategies (Velasco et al., 2018). Such perspective reflects a transition in our understanding of perception shifting from an independent view of the senses, toward a multisensory conception (Stein, 2012). In this multisensory framework it is accepted that input from one modality alters the perception of other modalities. This, together with long-term priors or associations, are constitutive aspects of perception (Lupyan and Clark, 2015; Piqueras-Fiszman and Spence, 2016). While taste and flavor are often used interchangeably in everyday language and even science, we here take the former to refer to sensations specifically arising from stimulation of the gustatory receptors, the latter as the perception arising from a combination of olfactory, gustatory and trigeminal sensations (see Spence et al., 2015 for a discussion on this distinction). Accordingly, there is evidence of many senses contributing to flavor perception (Stevenson and Mahmut, 2011; Spence, 2015; Piqueras-Fiszman and Spence, 2016). Particularly, colors of foods or beverages have shown to influence flavor perception and preference (DuBose et al., 1980; Clydesdale et al., 1992; Zellner and Durlach, 2003). The perceived sweetness of edibles, for example, has been enhanced by adding red color to a cherry-flavored solution; the more intense the color, the sweeter the flavor (Lavin and Lawless, 1998). Such effects are especially strong for colors and flavors with strong prior associations, like cherries that are associated with red and sweet or lemons with yellow and sour (Wieneke et al., 2018). This phenomenon has been refered to as a cross-modal correspondence (Spence, 2011), where a certain expectation about an attribute in one modality is transfered to another (e.g., redness/sweetness). These correspondences have been argued to be bidirectional (Deroy and Spence, 2013; Spence et al., 2015; Spence, 2019), such that, theoretically, both the redness of a cherry could enhance its perceived sweetness, and the sweetness could accentuate its perceived redness. Such correspondence might be a distinguishing factor between synesthesia and more universal multisensory associations (Deroy and Spence, 2013). Notably, this bidirectionality is not necessarily symetric, and may depend on individual reliance on sensory modalities (Deroy and Spence, 2013) and on the dominance of particular senses in humans (e.g., vision, which dominates many multisensory processes; Posner et al., 1976; Spence et al., 2001). Despite this assumption of bidirectionality in cross-modal correspondences, however, there is a large asymmetry between the number of studies investigating the influence of color on flavor perception, and those on how color might be influenced by flavor (Spence, 2019). Still, there are studies showing that certain basic tastes are indeed associated with colors (Saluja and Stevenson, 2018), as are linguistic references to taste (Spence et al., 2015), and that bidirectionality is present in other modalities (e.g., Mesfin et al., 2018). While relatively few endeavors have investigated bidirectional influences, even fewer—if at all— have aimed at investigating them in the context of cross-modal contrasts (i.e., contrasting the expectation from one modality to the experience in another modality; see Piqueras-Fiszman and Spence, 2015). For visual modulations of flavor in this context, for example, DuBose et al. (1980) showed that differently colored beverages were often misidentified, and Zellner and Durlach (2003) reported that beverages with the same flavor but varying in color were rated differently regarding their refreshment and liking. More recently, consistent findings of vision modulating flavor in virtual reality settings have been reported (Ammann et al., 2020). To our knowledge, however, no studies have investigated modulations on the other direction. We here thus aimed to study the potential bidirectionality of incongruent visuo-flavorous cross-modulations in an immersive and embodied mixed reality setup.

**FIGURE 1:**
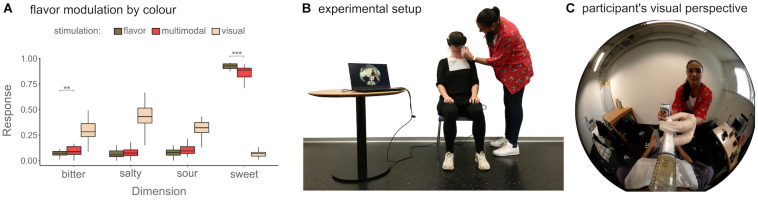
**(A)** Median and interquartile range for the participants’ mean flavor modulation by vision over all stimuli. Significance marks are indicated as follows: ***p < 0.001, **p < 0.01. **(B)** Shows the experimental setup during the multimodal contrasting condition for the color and flavor perception test (for illustration only, the real room corresponded to the one seen in **(C)**, and **(C)** the participants visual perspective during the experiment. Note that the seen liquid in **(C)** and the ingested liquid in **(B)** differed.

Across two tasks we examined how flavor perception could be modulated by vision and how color perception might be modulated by flavor, by comparing for each modality ratings after contrasting visuo-flavorous stimuli to ratings after unimodal stimuli. On a head mounted display (HMD), participants saw from the perspective of a male body a liquid being fed to them using a syringe, while they simultaneously were fed a liquid that contrasted the seen liquid (Figures 1B,C). To measure the effects of vision on flavor perception, participants judged the sweetness, sourness, bitterness and saltiness of the previously ingested liquid on visual analog scales (VAS). To measure how the ingested liquid’s flavor might affect the perceived color of the seen liquid, they selected the color of the previously seen liquid on a color wheel. In accordance with previous literature, we hypothesized that the flavor associated with the visual cues would bias the flavor of the liquid in the direction of such visual attributes in the contrast (multimodal) condition compared to a unimodal condition. We hypothesized this effect based on the chosen stimuli would be specifically for the sweetness and bitterness but not on sourness and saltiness dimensions. To assess the potential bidirectionality for cross-modal correspondences, we expected participants to judge the color of the beverage (in the multimodal condition) biased toward the color association of the ingested liquid (in the unimodal condition).

## Methods

### Participants

Thirty volunteers were recruited through a university mailing list considering the following criteria: being between 18 and 35 years old, not on diet, with normal or corrected-to-normal vision, no history of psychiatric, neurological or vestibular disease, nor food-related allergies, and not taking any medications. From the flavor perception part, two were excluded due to technical problems with the Oculus controller, resulting in a sample size of *N* = 28 (25 females; age: *M* = 23.39, *SD* = 4.47). From the color perception task, the same two plus an additional participant (due to feeling unwell) were excluded, resulting in a final sample size of *N* = 27 (24 females, age: *M* = 23.44, *SD* = 4.62). Participants provided informed consent, having been informed that the purpose of the study was to rate drinks, and received course credit.

### Materials

#### Virtual Reality Setup and Visual Stimulation

An Oculus CV1 head-mounted display (HMD) and the corresponding Oculus Touch controllers were used. Unity 2018 was used for displaying a 235-degrees pre-recorded video portraying the first-person perspective of a real person and presenting the tasks. The videos were filmed from the perspective of a male actor and lasted approximately 20 s each (see Supplementary Material for an example). Visual stimulation on the HMD was reactive to head movements. As visuo-tactile synchrony next to a matching posture and a first-person perspective have shown to enhance embodiment of the seen body (Maselli and Slater, 2013) regardless of the gender (Petkova and Ehrsson, 2008; Kilteni et al., 2015), at the beginning of each video participants were touched on the leg and simultaneously saw a corresponding touch on the virtual body. The VAS and color wheels to assess participants’ ratings were displayed on the HMD and answered with head movements and the Oculus controllers, respectively.

#### Flavor Stimulation

To guarantee reproducibility, we used artificial flavor products from Plusaroma^1^. One drop of every flavor was dissolved in 12 ml water, except for the peppermint flavor (a single drop for 24 ml). Such quantities were based on an explorative phenomenal assessment. A 10 ml Braun syringe was used to provide the liquid.

### Procedure

#### Experimental Design

**FIGURE 2:**
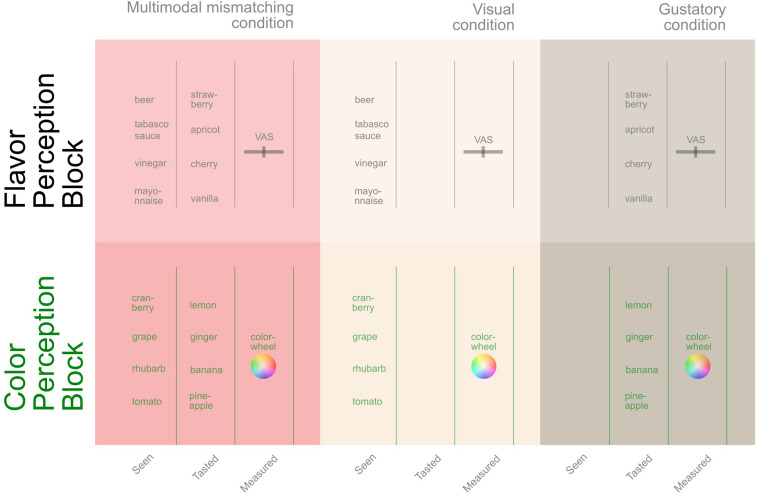
Overview of the experimental design of the here reported experiment showing both the flavor perception and the color perception blocks, as well as the stimuli presented for each task. The blocks were presented in a counterbalanced order.

The within-subject experiment was part of a larger study on visual/flavor conflicts on memory (not reported here, see Supplementary Material and the preregistered report at https://osf.io/xju3h/) and perception. The latter included both a color and a flavor perception block (see Figure 2) that were presented in a counterbalanced order and are reported here. In both blocks, participants saw the immersive videos from a first-person perspective in the HMD. The room, the furniture, their positioning, and the experimenter were identical in the real and the seen room for maximizing plausibility. Participants were required to match their body posture to that of the seen body in the headset and open their mouths whenever they saw the examiner approaching to feed them. The experimenter had previous extensive practice to ensure temporal synchrony between her actions and what participants saw. Given the location of the mouth from the visual perspective, slight temporal asynchronies when the syringe was close to the mouth were overlooked. Participants were asked to ingest the received liquid. Four flavorous and four visual stimuli were presented for the flavor perception task, and four alternative visual and flavorous stimuli for the color perception task. The contrasting stimuli for both tasks (described below) were chosen so that visual cues contrasted the flavor of the ingested product. To rule out any confounding variables, the stimuli were presented in a semi-counterbalanced manner, using four orders for each task. The overall procedure took approximately 1.5 h; the here reported part about 30 min.

#### Preparation

At the beginning of the overall procedure, participants seated on a chair positioned in the same location where the presented videos were previously recorded. After adjusting the HMD, to familiarize themselves with the tasks to follow they virtually saw the experimenter feeding them water while they were simultaneously fed water with a syringe. They were further instructed on selecting values on a VAS and options on a virtual color wheel before starting the experiment.

#### Flavor Perception Block

Participants saw on the HMD the examiner approaching to feed them while holding a liquid container (e.g., a beer can) and a syringe filled with the contained liquid (e.g., beer). Simultaneous to the feeding seen on the HMD, they were fed with 4 ml of an artificial flavor (flavorous stimuli), which tasted differently than the seen beverage (i.e., multimodal contrasting condition). The flavor stimuli were selected based on sweetness (i.e., apricot, vanilla, strawberry and cherry flavor), while the visual stimuli were associated with flavor dimensions other than sweetness (i.e., beer, vinegar, mayonnaise and tabasco sauce). After each exposure they rated the perceived flavor on a VAS (ranging from *not at all = 0* to *completely = 1*), which was displayed on the HMD after each condition with the questions *(“How sweet did you experience this liquid,” “How bitter did you experience this liquid,” “How sour did you experience this liquid,” “How salty did you experience this liquid”*).

To establish a baseline for each presented flavor, after each contrasting trial, they tasted the same flavor again but without any visual cue (i.e., unimodal gustation condition, presented with a black image on the HMD) followed by the same VAS. A baseline of the associated flavor for each visual stimulus was established by showing the same stimulus without any concomitant liquid (i.e., unimodal visual condition) followed by the VAS assessing the flavor of the seen liquid. See Figure 2 for the experimental design and stimulus pairings, and Figure 1 depicting how the stimuli were presented.

### Color Perception Block

The same experimental setup was used for this block, yet this time the contrasting visual versus flavorous stimuli were chosen to differ with regard to the typically associated colors, and we assessed color instead of flavor perception (Figure 2). For the visual stimuli red liquids were chosen (tomato, grape, rhubarb and cranberry juice) while the ingested liquids were associated with yellow colors (pineapple, banana, lemon and ginger flavor). After each exposure, we used a color wheel to rate color of the previously perceived liquid (see Saluja and Stevenson, 2018 for a similar measure). The measure was displayed on the HMD and captured the red, green and blue color dimensions.

To establish a color-association baseline for each liquid, after each contrasting trial, they tasted the same flavor again without any visual cue (i.e., unimodal gustation condition) and judged its color on the color wheel. The color baseline was established by presenting the identical visual stimuli without any liquid (i.e., unimodal vision condition) and selecting its color. Participants chose a color after each multimodal contrasting condition (*“Which color do you think the liquid you just saw is?”*), unimodal gustation condition (*“Which color do you think the liquid you just tasted is?”*) and unimodal vision condition (*“Which color do you think the liquid you just saw is?”*).

### Data Treatment

Data processing was performed using R 3.6.1 (R Core Team, 2020) and statistic tests using both R and JASP version 0.11.1. Alpha level was set at 0.05, or 95% confidence intervals. Data were tested for normality using Shapiro-Wilk tests and visual inspection. For parametric data the mean and standard deviation are reported as descriptive statistics, while for the non-parametric the median and interquartile are described. Rank-biserial correlation scores are reported as a measure of effect size. The averages of the four stimuli were coded by the mean of the four values for the multimodal and unimodal stimuli for each participant. For both tasks Wilcoxon signed-rank tests assessed whether the VAS responses and the selected colors differed between the multimodal and the unimodal condition of the manipulated modality. The baseline of the manipulating modality (i.e., vision when manipulating flavor perception and flavor when manipulating color perception) was used to confirm the direction of the perceptual change.

## Results

### Modulation of Flavor Perception Through Vision

After taking the average of the four trials, a Wilcoxon signed-rank test assessed whether the responses on each of the VAS scales for the multimodal and the unimodal flavors differed significantly. The test revealed a significant difference between the multimodal sweetness (*Mdn* = 0.88, *IQR* = 0.09) and unimodal gustation sweetness (*Mdn* = 0.93, *IQR* = 0.05; *W* = 61, *p* < 0.001, *rB* = −0.70) and also between the multimodal bitterness (*Mdn* = 0.09, *IQR* = 0.07) and unimodal gustation bitterness (*Mdn* = 0.07, *IQR* = 0.04; *W* = 332, *p* = 0.002, *rB* = 0.64). No significant differences between the multimodal sourness (*Mdn* = 0.09, *IQR* = 0.07) and unimodal gustation sourness (*Mdn* = 0.07, *IQR* = 0.05; *W* = 281, *p* = 0.08, *rB* = 0.38) nor between multimodal saltiness (*Mdn* = 0.07, *IQR* = 0.05) and unimodal gustation saltiness (*Mdn* = 0.06, *IQR* = 0.06; *W* = 247, *p* = 0.17, *rB* = 0.31) were found. Wilcoxon signed-rank tests were additionally performed between the unimodal flavorous- and multimodal stimuli for each of the four different trials (see Table 1).

**TABLE 1:** Wilcoxon signed-rank tests comparing the perceived flavor between the flavorous unimodal and the multimodal stimulation during the flavor modulation task (*N* = 28; ***p < 0.001, **p < 0.01, *p < 0.05).

Rated flavor		Sweetness multi vs.			Bitterness multi vs.			Sourness multi vs.			Saltiness multi vs.		
		unimodal			unimodal			unimodal			unimodal		
				
Visual item (packaging)	Flavorous item (artificial flavor)	*p*	*W*	*rB*	*p*	*W*	*rB*	*p*	*W*	*rB*	*p*	*W*	*rB*
Beer	Strawberry	0.007**	75.5	−0.60	0.03*	261.0	0.49	0.08	280.0	0.38	0.15	250.0	0.32
Tabasco sauce	Apricot	0.002**	60.0	−0.68	0.08	245.0	0.40	0.46	236.0	0.16	0.10	224.0	0.38
Vinegar	Cherry	0.003**	64.5	−0.66	0.04*	258.5	0.47	0.26	253.5	0.25	0.55	151.5	−0.14
Mayonnaise	Vanilla	<0.001***	43.5	−0.79	0.01**	237.0	0.35	0.16	248.5	0.31	0.04*	222.0	0.48

### Modulation of Color Perception Through Flavor

A Wilcoxon signed-rank test assessed whether the color values from the multimodal and the unimodal visual stimuli significantly differed on any of the color dimensions (RGB). For the average over the four stimuli, the test revealed no significant difference between the multimodal redness (*Mdn* = 0.64, *IQR* = 0.12) and unimodal vision redness (*Mdn* = 0.63, *IQR* = 0.07; *W* = 234, *p* = 0.29, *rB* = 0.24), between the multimodal greenness (*Mdn* = 0.24, *IQR* = 0.06) and unimodal vision greenness (*Mdn* = 0.23, *IQR* = 0.08; *W* = 203, *p* = 0.75, *rB* = 0.07), also between multimodal blueness (*Mdn* = 0.41, *IQR* = 0.09) and unimodal vision blueness (*Mdn* = 0.41, *IQR* = 0.10; *W* = 163, *p* = 0.55, *rB* = −0.14). See Table 2 for individual comparisons.

**TABLE 2:** Color values in red, green, blue format, and visualization of the perceived colors for the color modulation task, significance marks represent the comparison between the visual and the multimodal cues (**p < 0.01, *p < 0.05).

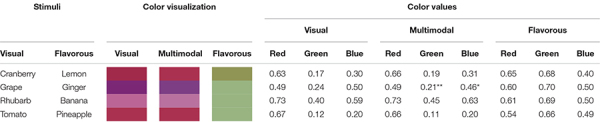

## Discussion

Using an embodied mixed reality setup, we investigated the influence of visual cues on flavor perception and that of flavor on color perception. Our results confirmed that visual information did modify the perceived flavor in the expected direction. As for the modulation of color by flavor, the data revealed no overall differences from baseline in the expected direction. These results thus show no evidence of bilateral cross-modal influences in the context of contrasting visuo-flavorous cues. We discuss the intricacies and considerations below.

### Modulation of Flavor by Vision

To investigate the potential modulation of flavor by visual expectancies, we combined visual cues stereotypically associated with bitterness alongside flavorous stimuli generally associated with sweetness. As expected, and in line with vast literature (Clydesdale et al., 1992; Piqueras-Fiszman and Spence, 2015; Spence, 2019), participants rated the perceived sweetness significantly lower in the contrasting than in the unimodal gustation condition, and the perceived bitterness higher in the contrasting compared to the unimodal condition. No differences were found for the mean of all the trials in the dimensions of sourness and saltiness, which accordingly were not targeted by the chosen stimuli. Thus, the direction of this effect seems clearly driven by the visual expectancy. These results are in line with theories suggesting that prior information about the edibles before consumption generates dominant expectations that modulate the experience of flavor (Lupyan and Clark, 2015; Spence, 2016) and confirm existing literature of cross-modal modulations with contrasting stimuli qualities (DuBose et al., 1980; Clydesdale et al., 1992; Zellner and Durlach, 2003). Our study extends previous literature by showing that vision-driven modulations of flavor can be created in embodied mixed reality settings, confirming that such modulations are independent of the visual-stimulation medium (see also Ammann et al., 2020).

### No Clear Modulation of Color by Flavor

Despite the dominant role of vision in human multisensory experience (Posner et al., 1976; Spence et al., 2001), various aspects of visual perception have shown to be modulated by other senses when there is a strong prior association between the two (e.g., Shams et al., 2000; Repp and Penel, 2002; Robinson and Sloutsky, 2013), and clear color associations to specific flavor and tastes have been reported (Spence et al., 2015; Saluja and Stevenson, 2018). However, much less is known about the potential of contrasting visuo-flavorous cues to modulate color perception (Spence, 2019). Participants ingested liquids associated with yellow or green while seeing a red liquid, to then judge the perceived color of the ingested liquid. There was no evidence of an effect of the contrasting visuo-flavorous cues on the perceived color in any of the color dimensions when taking the mean for all stimuli. The general null findings suggest no systematic modulation of color by flavor, further accentuating the dominant role of vision in human experience (Posner et al., 1976; but see also Hörberg et al., 2020). Interestingly, the colors associated with each of the flavorous stimuli (unimodal flavorous condition) were overly green. In fact, both green and yellow have been previously associated with sourness (Spence et al., 2015; Saluja and Stevenson, 2018), while our flavor selection was perhaps more heterogeneous (e.g., banana is not particularly salient in terms of sourness). Future studies are advised to limit the flavor selection of flavorous cues to a more homogeneous assortment in terms of basic taste dimensions to avoid any potential confounds (see Spence et al., 2015 for a distinction between associations in particular cases versus general cross-modal sensory features). As for the single item-pair showing significant changes (visual: grape, flavorous: ginger), the differences were in the opposite direction than expected (i.e., less green), thus not allowing us to make any conclusions for this change. A complete symmetry in bilaterality of cross-modulations was not expected, due to the general dominance of vision in our multisensory experience (Deroy and Spence, 2013; Spence, 2019) and its temporal precedence to flavor as found most natural conditions (see below). It has been theoretically argued that visual capture might be particularly strong in virtual reality due to the strength of the substitution of the visual field and optic flow (Roel Lesur et al., 2018), which could have further biased our results in favor of vision and might hinder generalization to other settings. Furthermore, color perception could potentially be modulated by here neglected aspects of flavor (such as textures or temperature) or natural flavors that might elicit a stronger association. Flavor is a multisensory construct and is not only defined by taste but also scents, textures, temperature, pain and sound (Yeomans et al., 2008). Here, we used water-based beverages, which limited the sensory stimulation of flavor to basic tastes and ortho- and retronasal stimulation of olfactory receptors (Koza et al., 2005), while other aspects were not modulated. Moreover, there might have been a potential floor and ceiling effect suggested by the comparatively high medians for sweetness and low for bitterness that could be accounted for by alternative, not so salient, stimuli or the ratio of artificial flavors and water used. Thus, the lack of evidence of color modulations here reported should not be taken as evidence for the general incapacity of flavor to manipulate color, but as a first step in elucidating the mechanics of potential bilaterality (or lack of it) in visuo-flavorous cross-modulations.

### Limitations of the Experimental Design

While our objective was to analyze the potential bilaterality of cross-modal influences in the context of contrasting visuo-flavorous stimuli, our assessment between modalities was itself not symmetric. A complete analogy between modalities could be impossible due to the ecological nature of our senses. For example, in ecological settings as much as in our experiment, vision tends to temporally precede gustation which might bias the interaction in the direction of the earlier modality (Piqueras-Fiszman and Spence, 2015). Thus, aiming for a closer analogy is not necessarily desired as it might imply a reduced ecological validity. As for our paradigm, this lack of symmetry is particularly salient in two ways. First, in that visual associations were generated mostly through packaging, and thus relied on high-level cultural associations and linguistic cues, whereas flavor associations were stimulated through artificial liquids that could have been not recognized. In this sense, explicit recognition might have played a role in the varying results between tasks. In general, both clearly recognizable cues as well as potentially not-recognizable ones have methodological advantages and limitations, including the cognitive processes involved (Spence, 2016). As a second asymmetry in our paradigm, flavor perception was linguistically assessed (through questionnaires) while color was directly judged in a color wheel. The complexity of assessing flavor and taste perception directly, however, is a general problem in the field (Saluja and Stevenson, 2018; Payne et al., 2021), and the primary aim of our study was to provide first evidence on a potential modulation of color through flavor. However, an issue with assessing flavor perception linguistically as we did (VAS) is the difficulty to convey the effect as a more tangible parameter. For example, it’s not clear how much sweeter in, say, estimated sugar spoons the perceptual change is. Future studies, however, might consider improving our design to account for these points.

Readers should note that the seen liquids may not have elicited a homogeneous expectation in terms of texture (e.g., beer and mayonnaise), an aspect that is not desirable as it might have, however, minimally—confounded our results (Yeomans et al., 2008). Lastly, this experiment was part of a larger study on visuo-flavorous conflicts (see Supplementary Material), which might have influenced our findings due to potential carry-over effects. However, between the first visuo-flavorous stimulation section of such study and the here reported experiment, a 30-min break was taken and different stimuli were used.

## Conclusion and Outlook

While bilateral associations flavor and color have been reported (Piqueras-Fiszman and Spence, 2015; Saluja and Stevenson, 2018; Spence, 2019), we could not confirm our hypothesis of flavor modulating color perception in the context of contrasting cues. Our mixed-reality experimental setting, however, did replicate findings of modulations of flavor by vision in such new setting (DuBose et al., 1980; Clydesdale et al., 1992; Lavin and Lawless, 1998; Zellner and Durlach, 2003). Further investigating the bilaterality of cross-modal influences remains important for a thorough understanding of our multisensory system, and hopefully more research on these lines will emerge in the coming years. Our use of embodied mixed reality technologies provides an easily replicable setup for manipulating and studying visuo-flavorous perception that might serve future endeavors. In fact, alterations of embodiment seem to be at the forefront of potential applications given their potential to alter cognition, affect and behavior (e.g., Dijkerman and Lenggenhager, 2018), but where chemo-senses have been vastly disregarded (Roel Lesur et al., 2020). Adding to the palette of cross-modulations that can be created in embodied mixed reality settings (Kilteni et al., 2015), our evidence of visual modulations of flavor might inform this growing field.

## Data Availability Statement

The datasets presented in this study can be found in online repositories. The names of the repository/repositories and accession number(s) can be found below: https://osf.io/xju3h.

## Ethics Statement

The studies involving human participants were reviewed and approved by the Ethics Committee of the Faculty of Arts and Social Sciences at the University of Zurich (Approval Number: 17.12.15). The patients/participants provided their written informed consent to participate in this study. Written informed consent was obtained from the individuals for the publication of any potentially identifiable images or data included in this article.

## Author Contributions

BL, MR, and LS designed the experiment, wrote the overall content of the article, and contributed to the statistical analysis and the results section. LS contributed with the data collection. MR programmed the tasks. All authors contributed to the article and approved the submitted version.

## Conflict of Interest

The authors declare that the research was conducted in the absence of any commercial or financial relationships that could be construed as a potential conflict of interest.

## Footnotes

1 www.plusaroma.ch
