# Supplementary material for: What Am I Drinking? Vision Modulates the Perceived Flavor of Drinks, but No Evidence of Flavor Altering Color Perception in a Mixed Reality Paradigm
Source: Front Psychol. 2021 Jul 20;12:641069. doi: 10.3389/fpsyg.2021.641069 (PMC8329379; doi:10.3389/fpsyg.2021.641069)
Supplement: Supplementary file 1 [file Table_1.DOCX]

Supplementary Material

**General Study Procedure**

The entire study was designed as a within-subject experiment that consisted of three blocks (see Figure 1). After the preparation, a first bock was designed for episodic memory encoding. Participants were presented with matching (8 stimuli) or contrasting (8 stimuli) visuo-gustatory liquids. After each stimulus they answered a question on similarity of the tasted and the seen liquid, a question as well as two questions on the embodiment of the visually seen body on a visual analogue scale. This encoding phase took about 30 minutes. Then participants did a 30 minutes break in which they a study irrelevant task. After this, in Block 2, we tested how many of the flavours and seen liquid of Block 1 were correctly recognized. For this they tasted both previously seen/tasted or previously unseen/untasted liquid and answered with a forced choice response. In the third block color and taste perception were tested, which is the content of the present main manuscript. .

Figure 1. A within-subject experiment with three blocks was performed: 1) episodic memory encoding and embodiment measures; 2) late memory recall; 3) taste and color perception
